# Supplementary material for: Retrospective challenges to pre-exposure prophylaxis (PrEP) use among people living with HIV—A qualitative analysis using the COM-B framework
Source: PLoS One. 2026 Feb 13;21(2):e0325871. doi: 10.1371/journal.pone.0325871 (PMC12904379; doi:10.1371/journal.pone.0325871)
Supplement: S1 Text — (DOCX) [file pone.0325871.s001.docx]

# Supporting information 1 (S1) Topic guide

Demographics

- Date of Birth and current age
- Date of + diagnosis
- Date of last - diagnosis
- Ethnicity
- Gender
- Country of birth, if born abroad, how long been in the UK
- Were they working at time of diagnosis, and if so, what was their job *(as an indicator for socio-economic status).*
- Sexual orientation *(this information is provided on SHARE/LEADS form, but good to confirm. Not necessary to ask at this stage as questions are addressed later in interview).*

**NB – The following questions may not necessary follow this order, as it will depend on the interviewee and the information they provide, so need to be flexible!**

How and why were they diagnosed with HIV?

- Reason for testing *(what motivated them)*
- What do they think was their Likely exposure event
- For recent seroconverters- Are they regular testers *(if yes, why, where and how often)*

Testing history- for late diagnoses only

- Have they tested previously? *(if yes, when and why?)*
- Have they ever been offered a test and declined? *(if yes, why?)*
- If never tested before, did they ever consider testing? *(if no, why not? If yes, why didn’t they?)*

Ideas about HIV and HIV risk

- HIV knowledge and attitudes
- Perception of own risk *(changed attitude to risk?)*

Actual HIV risk

- Actual risk
- Number of partners *(partners from abroad - where, partners abroad – where, paid for sex?)*
- Type of partners *(regular/casual)*
- How meet *(internet use and which dating sites)*
- Type of sex, *(top/bottom, aware of sero-positioning)*
- Sexualised drug use *(sex parties, chemsex, which drugs)*
- Previous STIs
- Conversations with partners before sex *(talk about condom use, PrEP, HIV diagnosis/status, on treatment)*
- Considered taking PrEP themselves

Behaviors and experiences of Prevention

- Know much about preventing HIV?
- Experiences and attitudes of/to prevention
- Prevention options *(condoms, negotiated safer sex, sero-positioning, PrEP (continuous or as required), PEP, HIV status)*
- Discuss these options with partners?
- Awareness, attitudes, experience of PrEP/ PEP, partners on treatment (changed attitude to risk?)
- Now HIV+, how do they feel about diagnosis now

Experiences of Services and Campaigns

- Experiences of sexual health services and HIV testing *(inc. contraceptives, STIs)*
- For late diagnoses- probe for knowledge around where possible to test for HIV/HIV testing sites
- Service improvement and recommendations *(what would have helped them)*
- For late diagnoses- ways to make it easier to get a test
- Broader health care services, and use of them *(GPs, internet and which sites, and how judge them)*
- HIV health Promotion *(what seen or not, what was good/not good, gaps, how do they judge, what would have helped them).* For late diagnoses- probe on testing promotion
- Why they looked for information

Potential for Partner Notification

- Informed any partners *(they did or clinic informed)*
- Disclosed to others *(family, friends, new sexual partners)*
- What could clinics do to support around partner notification
- For late diagnoses- have they ever received a partner notification for HIV *(if yes, what was the action after this?)*
