# Supplementary material for: Retrospective challenges to pre-exposure prophylaxis (PrEP) use among people living with HIV—A qualitative analysis using the COM-B framework
Source: PLoS One. 2026 Feb 13;21(2):e0325871. doi: 10.1371/journal.pone.0325871 (PMC12904379; doi:10.1371/journal.pone.0325871)
Supplement: S2 Text — (DOCX) [file pone.0325871.s002.docx]

**Supporting information 2 (S2) Codebook**

| **COM-B domain** | **Decision rule – theoretical (aided by Theoretical Domains Framework domains)** | **Decision rule – informed by the data** | **Examples (B = barrier; F = facilitator)** |
| --- | --- | --- | --- |
| **Capability** - attribute of a person that makes behaviour possible or facilitates it; includes mental functioning and a person’s physique and musculoskeletal functioning   - Psychological capability (mental functioning (e.g., understanding and memory) - Physiological capability (person’s physique and musculoskeletal functioning) | - Knowledge - Memory, attention, and decision processes - Skills - Behavioural regulation   If it doesn’t concern any of these (and does not concern the other domains => miscellaneous data)  Only code participant’s immediate barriers/ facilitators (assumptions about barriers/ facilitators etc, consider under recommendations) | - Understanding of HIV preventions (including how to access it)   - Do not code accounts about actual or anticipated access issues 🡪 Opportunity/ Motivation   - Do not code opinions and beliefs 🡪 Motivation - Lack of understanding - Ability (skills and physical) to engage with HIV prevention and lack thereof   - Drugs if they impair physical state (if influenced by others 🡪 Opportunity) - Overarching   Any of the above that does not explicitly refer to PrEP but that could still impact it | *So, I knew of, I know people some people were on [PrEP], but I wouldn’t have had a clue how to get it. You know, how it starts, how, you know. […] Whether [PrEP is available from the] NHS, whether I would have been old enough to have it. I wouldn’t. I don’t know.* (B)  *No. To be honest, [I did] not much [know about HIV before the diagnosis].. . A little bit. ..and that’s only mainly because of TV, obviously there's a lot more talk about it than there has been in the past... shows and things, as I said.* (B) |
| **Opportunity -** attribute of a person that makes behaviour possible or facilitates it; includes inanimate parts of the environment, system, and time, as well as other people and organizations   - Social opportunity (other people and organizations, including culture and social norms) - Physical opportunity (inanimate parts of environmental system, time (financial and material resources) | - Social influences   - Social pressure, norms, social support - Environmental context and resources   - Organizational culture/ climate   If it doesn’t concern any of these (and does not concern the other domains => miscellaneous data)  Only code participant’s immediate barriers/ facilitators (assumptions about barriers/ facilitators etc, consider under recommendations) | - Interaction with others (sexual partners, health care professionals) that influences the use/ consideration of HIV prevention   - Including missed opportunities where these constituted barriers (do not code if mentioned as a hypothetical recommendation 🡪 Recommendation)   - Interaction with others that lead to reinforcement 🡪 Motivation   - Actual negative/ positive treatment by others (do not code anticipated ones 🡪 Motivation) - Perception of social norms for/ against using HIV prevention - Access to HIV prevention (including any logistical issues) - Overarching   Any of the above that does not explicitly refer to PrEP but that could still impact it | *[…] one [nurse] there which was taking my blood once for my test, she says, ‘Oh, you know, this PrEP is just for the people that they, you know, having unprotected sex. And if you're not really doing that, they won’t, they won’t really offer you that at least now because it's so limited’ right before they become free under NHS.* (B)  *I was still, you know, I was literally on my own. I didn’t have any other gay friends […] So, it’s quite hard to find the information out there. ’You’ve got to go and look for yourself.* (B) |
| **Motivation -** aggregate of mental processes that energizes and directs behaviour; includes conscious thought as well as habitual, instinctive, and affective processes   - Reflective motivation (conscious thought, i.e., plans and evaluations) - Automatic motivation (habitual and instinctive including desires) | - Reinforcement (positive and negative) - Emotions - Social/ professional role and identity - Beliefs about capabilities - Optimism - Beliefs about consequences - Intentions - Goals   If it doesn’t concern any of these (and does not concern the other domains => miscellaneous data)  Only code participant’s immediate barriers/ facilitators (assumptions about barriers/ facilitators etc, consider under recommendations) | - Reinforcement as a consequence of previous infection and use of HIV prevention (or lack thereof) or interaction with others - Pleasure or lack thereof when using HIV prevention - Lifestyle (habitual and concerns) - Match with identities (or lack thereof)   - Do not code ‘internalized social norms’ 🡪 Opportunity - Attitudes towards HIV prevention - Beliefs about the ability to use HIV prevention - False optimism (related to reinforcement) not using HIV prevention/ not acquiring an infection - Beliefs about what the consequences are of using HIV prevention (positive and negative)   - Risk perceptions, including trusting others (where participants were actively influenced by others 🡪 Opportunity)   - Anticipated access issues (actual access 🡪 Opportunity)   - Anticipated negative treatment (actual negative treatment 🡪 opportunity) - Intentions to use HIV preventions - Goals that may hinder or facilitate the use of HIV prevention - Overarching   Any of the above that does not explicitly refer to PrEP but that could still impact it | *I didn't feel any of these things were part of my life quite arrogantly […] you know, when you're [between 50 to 64 years] and you think, you know people, you may still have a few sexually transmitted infections as I [said?] to you. I just for when they report being single man and gay, you know, I [could’ve?], locked myself away and but which I didn’t want to do, I suppose, I was going to get something but* *never expecting, don't get HIV.* (B)  *I think we did talk about it and that's why I was on PrEP, etc., and I did everything I could to remain safe.* (F) |
